# Impact of Vaccination Status on Outcome of Patients With COVID‐19 and Acute Ischemic Stroke Undergoing Mechanical Thrombectomy

**DOI:** 10.1161/JAHA.123.031816

**Published:** 2024-04-19

**Authors:** Cornelius Deuschl, Lukas Goertz, Christoph Kabbasch, Martin Köhrmann, Christoph Kleinschnitz, Ansgar Berlis, Christoph Johannes Maurer, Iris Mühlen, Bernd Kallmünzer, Matthias Gawlitza, Daniel P. O. Kaiser, Joachim Klisch, Donald Lobsien, Daniel Behme, Maximilian Thormann, Fabian Flottmann, Laurens Winkelmeier, Elke Ruth Gizewski, Lukas Mayer‐Suess, Markus Holtmannspoetter, Christoph Moenninghoff, Martin Schlunz‐Hendann, Dominik Grieb, Christophe T. Arendt, Ferdinand O. Bohmann, Jens Altenbernd, Yan Li, Ulrich Sure, Ruben Mühl‐Benninghaus, Thomas Rodt, Kai Kallenberg, Alexandru Durutya, Mohamed Elsharkawy, Christian‐Paul Stracke, Mathias Gerhard Schumann, Alexander Bock, Omid Nikoubashman, Martin Wiesmann, Hans Henkes, Sebastian Dolff, Aydin Demircioglu, Michael Forsting, Hanna Styczen

**Affiliations:** ^1^ Institute for Diagnostic and Interventional Radiology and Neuroradiology University Hospital Essen Essen Germany; ^2^ Department of Diagnostic and Interventional Radiology University Hospital Cologne Cologne Germany; ^3^ Department of Neurology and Center for Translational Neurosciences and Behavioral Sciences University Hospital Essen Essen Germany; ^4^ Department of Diagnostic and Interventional Neuroradiology University Hospital Augsburg Augsburg Germany; ^5^ Department of Neuroradiology University of Erlangen‐Nuremberg Erlangen Germany; ^6^ Department of Neurology University of Erlangen‐Nuremberg Erlangen Germany; ^7^ Faculty of Medicine, Institute and Policlinic of Neuroradiology, University Hospital Carl Gustav Carus Technische Universität Dresden Dresden Germany; ^8^ Department of Neuroradiology University Hospital Leipzig Leipzig Germany; ^9^ Department of Diagnostic and Interventional Radiology and Neuroradiology Helios General Hospital Erfurt Erfurt Germany; ^10^ Department of Neuroradiology University Hospital Magdeburg Magdeburg Germany; ^11^ Department of Radiology University Hospital Magdeburg Magdeburg Germany; ^12^ Department of Diagnostic and Interventional Neuroradiology University Medical Center Hamburg‐Eppendorf Hamburg Germany; ^13^ Department of Neuroradiology Medical University Innsbruck Innsbruck Austria; ^14^ Department of Neurology Medical University Innsbruck Innsbruck Austria; ^15^ Department of Neuroradiology Nuremberg General Hospital Nuremberg Germany; ^16^ Department of Radiology, Neuroradiology and Nuclear Medicine Johannes Wesling University Hospital, Ruhr University Bochum Bochum Germany; ^17^ Department of Radiology and Neuroradiology Klinikum Duisburg–Sana Kliniken Duisburg Germany; ^18^ Department of Diagnostic and Interventional Neuroradiology Medical School Hannover Hannover Germany; ^19^ Institute of Neuroradiology, University Hospital Goethe University Frankfurt am Main Germany; ^20^ Department of Radiology and Neuroradiology Gemeinschaftskrankenhaus Herdecke Herdecke Germany; ^21^ Department of Neurosurgery and Spine Surgery University Hospital of Essen Essen Germany; ^22^ Department of Radiology Klinikum Lueneburg Lueneburg Germany; ^23^ Department of Neuroradiology Klinikum Fulda Fulda Germany; ^24^ Clinic for Radiology University Hospital Muenster Muenster Germany; ^25^ Department of Neuroradiology Vivantes Klinikum Neukoelln Berlin Germany; ^26^ Department of Diagnostic and Interventional Neuroradiology University Hospital, Rheinisch‐Westfälische Technische Hochschule Aachen University Aachen Germany; ^27^ Clinic for Neuroradiology Klinikum Stuttgart Stuttgart Germany; ^28^ Department of Infectious Diseases, West German Centre of Infectious Diseases University Hospital Essen Essen Germany

**Keywords:** acute ischemic stroke, COVID‐19, COVID‐19 vaccination, mechanical thrombectomy, Cerebrovascular Disease/Stroke

## Abstract

**Background:**

Data on impact of COVID‐19 vaccination and outcomes of patients with COVID‐19 and acute ischemic stroke undergoing mechanical thrombectomy are scarce. Addressing this subject, we report our multicenter experience.

**Methods and Results:**

This was a retrospective analysis of patients with COVID‐19 and known vaccination status treated with mechanical thrombectomy for acute ischemic stroke at 20 tertiary care centers between January 2020 and January 2023. Baseline demographics, angiographic outcome, and clinical outcome evaluated by the modified Rankin Scale score at discharge were noted. A multivariate analysis was conducted to test whether these variables were associated with an unfavorable outcome, defined as modified Rankin Scale score >3. A total of 137 patients with acute ischemic stroke (48 vaccinated and 89 unvaccinated) with acute or subsided COVID‐19 infection who underwent mechanical thrombectomy attributable to vessel occlusion were included in the study. Angiographic outcomes between vaccinated and unvaccinated patients were similar (modified Thrombolysis in Cerebral Infarction ≥2b: 85.4% in vaccinated patients versus 86.5% in unvaccinated patients; *P*=0.859). The rate of functional independence (modified Rankin Scale score, ≤2) was 23.3% in the vaccinated group and 20.9% in the unvaccinated group (*P*=0.763). The mortality rate was 30% in both groups. In the multivariable analysis, vaccination status was not a significant predictor for an unfavorable outcome (*P*=0.957). However, acute COVID‐19 infection remained significant (odds ratio, 1.197 [95% CI, 1.007–1.417]; *P*=0.041).

**Conclusions:**

Our study demonstrated no impact of COVID‐19 vaccination on angiographic or clinical outcome of COVID‐19–positive patients with acute ischemic stroke undergoing mechanical thrombectomy, whereas worsening attributable to COVID‐19 was confirmed.

**Nonstandard Abbreviations and Acronyms**
AIS: acute ischemic stroke
IVT: intravenous thrombolysis
mRS: modified Rankin Scale
MT: mechanical thrombectomy
mTICI: modified Thrombolysis in Cerebral Infarction
NIHSS: National Institutes of Health Stroke Scale
NoVAX: not vaccinated
sICH: symptomatic intracranial hemorrhage
VAX: previously vaccinated

**Clinical Perspective**

**What Is New?**

- COVID‐19 vaccination did not result in a better clinical outcome in patients with acute ischemic stroke undergoing mechanical thrombectomy.

**What Are the Clinical Implications?**

- This study confirms a poor clinical outcome for patients with COVID‐19 and an acute ischemic stroke undergoing mechanical thrombectomy.

The COVID‐19 pandemic has affected millions of people worldwide, leading to unprecedented challenges for health care systems. Among the many challenges posed by the virus is the increased risk of complications, including acute ischemic stroke (AIS), with an incidence of 6% to 46% among hospitalized patients with COVID‐19.^1^, ^2^ In addition, recent studies of large series of patients with COVID‐19 infection have reported a devastating clinical outcome of patients with AIS attributable to large‐vessel occlusion treated with mechanical thrombectomy (MT), with mortality rates up to 31%.^3^, ^4^

Since the end of 2020, several vaccines, such as Comirnaty (BNT162b2, Pfizer‐BioNTech), Spikevax (mRNA‐1273, Moderna), Vaxzevria (ChAdOx1 nCoV‐19, AstraZeneca), and Jcovden (Ad26.COV‐2.S, Johnson & Johnson/Janssen), have been approved for emergency use in response to the COVID‐19 pandemic, with growing evidence of the safety and efficacy of vaccination against the SARS‐CoV‐2.^5^ Clinical trials have shown that the vaccines are effective in reducing the severity of COVID‐19 (duration and severity of symptoms), as well as the incidence of hospitalization, intensive care unit admission, and mortality rates.^6^, ^7^, ^8^, ^9^, ^10^ However, precise analyses of the preventive role of vaccination in patients with COVID‐19 experiencing AIS because of large‐vessel occlusion are still lacking.

Our study focuses on how vaccination status affects angiographic and clinical outcomes in patients with COVID‐19 and AIS undergoing MT and provides insight into the potential benefits of vaccination in this vulnerable patient population.

## Methods

The data that support the findings of this study are available from the corresponding author on reasonable request. We conducted a retrospective study of patients with COVID‐19 and AIS attributable to large‐ or medium‐vessel occlusion who were treated with MT at 20 tertiary care centers in Germany and Austria between January 2020 and January 2023. All patients receiving MT because of vessel occlusions, including distal internal cerebral artery, middle cerebral artery (M1, M2, and M3), anterior cerebral artery (A1 and A2), basilar artery, and posterior cerebral artery (P1 and P2), were identified. Documentation of vaccination status of all patients included unvaccinated and vaccinated patients with at least 1 vaccine: Comirnaty (BNT162b2, Pfizer‐BioNTech), Spikevax (mRNA‐1273, Moderna), Vaxzevria (ChAdOx1 nCoV‐19, AstraZeneca), and Jcovden (Ad26.COV‐2.S, Johnson & Johnson/Janssen).

A confirmed diagnosis of COVID‐19 was defined as a positive laboratory result for SARS‐CoV‐2 by high‐throughput sequencing or reverse transcription–polymerase chain reaction assay of nasal or oropharyngeal swab specimens.

Baseline characteristics, including respiratory status during hospitalization, technical features, complications, angiographic variables, and clinical outcomes were noted. The cause of the occlusion was based on the Trial of Org 10172 in Acute Stroke Treatment (TOAST) classification. There were no limitations on procedural characteristics, including the use of different thrombectomy techniques and intra‐arterial thrombolysis, which were left to the attending neuroradiologist's discretion. Endovascular treatment was performed with approved MT devices, using stent retrievers, large‐bore aspiration catheters, or a combination of both.

Reperfusion was measured by the modified Thrombolysis in Cerebral Infarction (mTICI) scale score. The clinical efficacy outcome was the rate of functional independence as measured by the modified Rankin Scale (mRS) score and defined as 0 to 2 at discharge. All National Institutes of Health Stroke Scale (NIHSS) and mRS grades were assessed by a consultant neurologist. Postinterventional symptomatic intracranial hemorrhage (sICH) was graded according to the ECASS (European Cooperative Acute Stroke Study) criteria.^11^

According to the guidelines of the respective local ethics committees, ethical approval was given when necessary for this anonymous retrospective study, which was conducted in accordance to the Declaration of Helsinki. A patient's consent for treatment was obtained according to the individual institutional guidelines. Because of the retrospective nature of the study, additional informed consent was deemed unnecessary.

### Statistical Analysis

Qualitative parameters are presented as numbers and percentages and compared with the χ^2^ and the Fisher exact test, when appropriate. Ordinal and quantitative parameters are presented as median and interquartile range, unless otherwise indicated. Group comparisons of these parameters were performed with Mann‐Whitney *U* test or the 2‐sided Student *t*‐test, when appropriate.

The primary outcome of interest was the mRS score at discharge, where a score between 0 and 2 was to be considered to be favorable. A multivariable analysis adjusted for confounders was conducted to test the association of the variables (age, sex, stroke onset, intravenous thrombolysis [IVT], vaccination status, occlusion site [M1, M2, M3, A1, A2, or basilar artery], patients with acute COVID‐19, respiratory status of patients with acute COVID‐19 [none versus ventilation and none versus intubation], TOAST cardioembolic, TOAST, small‐vessel occlusion, tandem occlusion, baseline aspect, NIHSS score at admission, mRS score at pretreatment, final TICI [2a], final TICI [2b], final TICI [3], final TICI [1], groin to final recanalization, anesthesia [analgosedation], complications [subarachnoid hemorrhage and sICH], other complications, atrial fibrillation, arterial hypertension, diabetes, dyslipidemia, and smoker) with this outcome. For this, a smoothed ridge regression with a logit‐link function was used. Ridge regression was used because of its ability to produce robust estimates, especially when dealing with smaller sample sizes.^12^ Missing values were not imputed. Calculations were performed using SPSS software, version 25 (IBM SPSS Statistics for Windows, IBM Corp, Armonk, NY), and R, version 4.3 (R Foundation for Statistical Computing, Vienna, Austria). *P*<0.05 was considered statistically significant.

## Results

A total of 137 of 6163 screened patients (2.2%) from 20 tertiary stroke centers with COVID‐19 infection and known vaccination status were treated with MT because of vessel occlusions between January 2020 and January 2023 (Figure).

**Figure 1:**
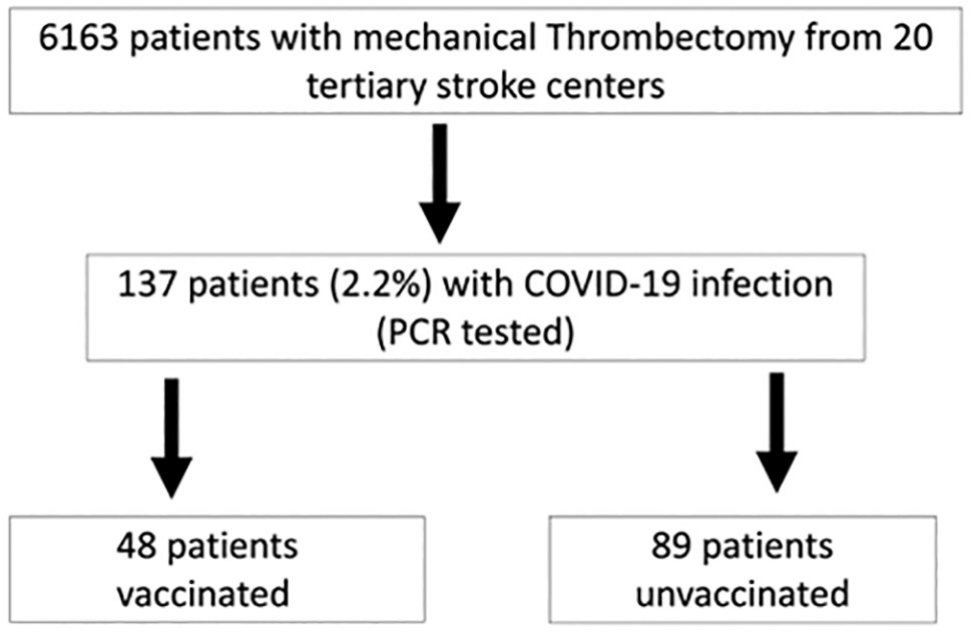
Flowchart of the study population. PCR indicates polymerase chain reaction.

### Baseline Characteristics

Of the 137 patients, 48 were previously vaccinated (VAX) and 89 were not vaccinated (NoVAX). Patient baseline characteristics are reported in Table 1.

**Table 1:** Baseline Characteristics of Unvaccinated and Vaccinated Patients With COVID‐19 Infection Undergoing MT

Variable	Unvaccinated patients with COVID‐19 treated by MT (n=89)	Vaccinated patients with COVID‐19 treated by MT (n=48)	*P* value
Demographics			
Age, mean±SD, y	71.7±15.5	72.2±13.5	0.853
Sex (male)	53 (59.6)	33 (68.8)	0.288
COVID‐19 infection			
Acute	67 (75.3)	31 (64.6)	0.186
Subsided	22 (24.7)	17 (35.4)	
Respiratory status during hospitalization			
No respiratory distress	34/67 (50.7)	21/31 (67.7)	0.115
Acute respiratory failure requiring noninvasive ventilation	14/67 (20.9)	3/31 (9.7)	0.253
Acute respiratory failure requiring intubation	19/67 (28.4)	7/31 (22.6)	0.547
Duration of invasive ventilation, median (range), d	4 (1–42)	8 (7–21)	0.371
Medical history			
Arterial hypertension	62 (69.7)	36 (75.0)	0.509
Atrial fibrillation	34 (38.2)	26 (54.2)	0.072
Diabetes	26 (29.2)	11 (22.9)	0.428
Dyslipidemia	28 (31.5)	14 (29.2)	0.781
Smoking	10 (11.2)	6 (12.5)	0.789
Stroke characteristics			
mRS score prestroke ≤2	55 (61.8) (n=84)	32/46 (69.6) (n=46)	0.372
Baseline NIHSS score, median (IQR)	17 (12–20) (n=88)	17.5 (13–22)	0.605
Baseline ASPECTS, median (IQR)	9 (8–10) (n=86)	9 (7–10) (n=47)	0.973
Wake‐up stroke	38 (42.7)	14/47 (29.8)	0.141
Intravenous thrombolysis	37 (41.6)	23 (47.9)	0.475
Onset‐to‐groin time, median (IQR), min	202.5 (146–305)	241 (180–285)	0.710
Site of occlusion			
Distal ICA	20 (22.5)	9 (18.8)	0.611
MCA M1	37 (41.6)	26 (54.2)	0.158
MCA M2	23 (25.8)	3 (6.3)	0.006
MCA M3	0 (0)	1 (2.1)	0.350
ACA	1 (1.1)	1 (2.1)	>0.999
BA	8 (9.0)	6 (12.6)	0.517
PCA	0 (0)	2 (4.2)	0.121
TOAST			0.709
Large‐artery sclerosis	14 (15.7)	9 (18.8)	0.641
Cardioembolic	40 (44.9)	25 (52.1)	0.425
Small‐vessel occlusion	2 (2.2)	1 (2.1)	>0.999
Other (infectious)	6 (6.7)	1 (2.1)	0.421
Undetermined	27 (30.3)	12 (25.0)	0.509
Reocclusion within 30 d	5 (5.6)	1 (2.1)	0.665
Tandem occlusion	14 (15.7)	4 (8.3)	0.293

Data are given as number (percentage) or number/total (percentage) unless otherwise indicated. ACA indicates anterior cerebral artery; ASPECTS, Alberta Stroke Program Early CT [Computed Tomography] Score; BA, basilar artery; ICA, internal carotid artery; IQR, interquartile range; MCA, middle cerebral artery; mRS, modified Rankin Scale; MT, mechanical thrombectomy; NIHSS, National Institutes of Health Stroke Scale; PCA, posterior cerebral artery; and TOAST, Trial of Org 10172 in Acute Stroke Treatment.

A total of 31 of 48 patients (64.6%) in the VAX group and 67 of 89 patients (75.3%) in the NoVAX group had a laboratory‐confirmed diagnosis of acute COVID‐19 infection, whereas 17 of 48 patients (35.4%) in the VAX group and 22 of 89 patients (24.7%) in the NoVAX group presented after subsided COVID‐19 infection. Most patients in the VAX group had been vaccinated with Comirnaty (BNT162b2, Pfizer‐BioNTech; 30/38, 62.5%), followed by Jcovden (Ad26.COV‐2.S, Johnson & Johnson/Janssen; 4/48, 8.3%) and ChAdOx1‐S (AstraZeneca; 2/48, 4.2%). In 12 of 48 patients (25%), the specific vaccine type was not documented.

Acute respiratory failure requiring noninvasive ventilation or intubation was more frequent in the NoVAX group compared with the VAX group (Table 1). Atrial fibrillation was more frequent in the VAX group than in the NoVAX group, but without statistical significance. Stroke characteristics did not differ significantly between groups: median baseline NIHSS score and Alberta Stroke Program Early CT [Computed Tomography] Score were 17 and 9 in both groups, respectively. The rate of pretreatment functional independence (mRS score, ≤2) was 69.6% in the VAX group and 61.8% in the NoVAX group. Vessel occlusion was localized in the anterior circulation in most patients, most commonly in M1 (Table 1). Occlusions in M2 were significantly more frequent in the NoVAX group (Table 1).

### Procedural and Functional Outcome

Most MTs were performed under general anesthesia in 129 of 137 patients (94.1%), followed by conscious sedation in 8 of 137 patients (5.8%). The median number of thrombectomy maneuvers was 2 (interquartile range, 1–3), and the median time interval from groin to final reperfusion was 38 minutes (interquartile range, 25–71 minutes). In most procedures, the first‐pass technique was a combined approach with aspiration and stent‐retriever thrombectomy (80/137, 58.3%), followed by aspiration thrombectomy in 50 of 137 patients (36.5%) and stent‐retriever thrombectomy in 2 of 137 patients (1.5%). In 1 case, spontaneous recanalization occurred; and in 4 cases, the thrombus localization could not be reached.

Angiographic outcomes did not differ between groups (Table 2): successful reperfusion (mTICI ≥2b) was achieved in 41 of 48 (85.4%) vaccinated and in 77 of 89 (86.5%) unvaccinated patients (*P*=0.859). Complete reperfusion (mTICI 3) was reached in 19 of 48 (39.6%) vaccinated and in 39 of 89 (43.8%) unvaccinated patients (*P*=0.632). Complications occurred in 14 of 48 (29.2%) vaccinated and in 12 of 89 (13.5%) unvaccinated patients (*P*=0.073). Patients in the VAX group had a significantly higher rate of sICH (7/48, 14.6%) than patients in the NoVAX group (4/89, 4.5%; *P*=0.05). The rate of subarachnoid hemorrhage and other complications (such as iatrogenic intra‐arterial dissection without hemodynamic relevance) was 14.6% (7/48) in the VAX group compared with 9% (8/89) in the NoVAX group.

**Table 2:** Angiographic and Clinical Outcomes of Unvaccinated and Vaccinated Patients With COVID‐19 Infection Undergoing MT

Variable	Unvaccinated patients with COVID‐19 treated by MT (n=89)	Vaccinated patients with COVID‐19 treated by MT (n=48)	*P* value
Angiographic outcomes			
Successful reperfusion (mTICI ≥2b)	77 (86.5)	41 (85.4)	0.859
Complete reperfusion (mTICI 3)	39 (43.8)	19 (39.6)	0.632
First‐pass successful reperfusion (mTICI ≥2b)	37/37 (100)	21/21 (100)	1.0
First‐pass complete reperfusion (mTICI 3)	22/37 (59.5)	9/21 (42.9)	0.223
Groin puncture–to–reperfusion time, median (IQR), min	35 (24–60)	42.5 (27–81)	0.323
No. of passes, median (IQR)	2 (1–3)	2 (1–4)	0.288
Procedure‐related complications			
sICH	4 (4.5)	7 (14.6)	0.050
SAH	7 (7.9)	6 (12.5)	0.377
Other	1 (1.1)	1 (2.1)	>0.999
Clinical outcomes			
NIHSS score at discharge, median (IQR)	10 (4–12)	15.5 (5–42)	0.458
mRS score ≤2 at discharge	18/86 (20.9)	10/43 (23.3)	0.763
Mortality at discharge	25/86 (29.1)	13/43 (30.2)	0.891

Data are given as number (percentage) or number/total (percentage) unless otherwise indicated. IQR indicates interquartile range; mRS, modified Rankin Scale; MT, mechanical thrombectomy; mTICI, modified Thrombolysis in Cerebral Infarction; NIHSS, National Institutes of Health Stroke Scale; SAH, subarachnoid hemorrhage; and sICH, symptomatic intracranial hemorrhage.

Vaccinated and unvaccinated patients with stroke had a similar prognosis at discharge: the rate of functional independence (mRS score, ≤2) was 23.3% in the VAX group and 20.9% in the NoVAX group. The median NIHSS score at discharge was higher in the VAX group than in the NoVAX group, although without statistical significance (15.5 [interquartile range, 5–42] VAX versus 10 [interquartile range, 4–12] NoVAX; *P*=0.458). The mortality rate was 30.2% (13/43) in the VAX group and 29.1% (35/86) in the NoVAX group (*P*=0.891). Clinical outcome at discharge was not documented in 5 vaccinated and in 3 unvaccinated patients.

In a subgroup analysis, unvaccinated patients with acute COVID‐19 infection and respiratory failure requiring noninvasive ventilation or intubation had a mortality rate of 46% compared with 40% in vaccinated patients (15/33 NoVAX versus 4/10 VAX; *P*=0.760).

### Multivariable Analysis

We performed a multivariable analysis to identify predictors associated with an unfavorable outcome (mRS score, 3–6) at discharge using an induced smooth ridge regression (Table 3). In the analysis, factors potentially associated with an unfavorable outcome were: acute COVID‐19 infection (odds ratio [OR], 1.43 [95% CI, 1.07–1.91]; *P*=0.016), acute respiratory failure requiring intubation in acute COVID‐19 infection (OR, 1.35 [95% CI, 1.08–1.69]; *P*=0.010), higher baseline Alberta Stroke Program Early CT Score (OR, 0.83 [95% CI, 0.69–0.98]; *P*=0.028), and higher pretreatment functional independence (OR, 1.23 [95% CI, 1.05–1.46]; *P*=0.010).

**Table 3:** Results of the Multivariable Analysis for an Unfavorable Outcome

Variable	Estimate	OR	95% CI	*P* value
Age	0.00	1.00	0.97–1.03	0.979
Sex (male vs female)	−0.04	0.96	0.72–1.28	0.788
Unknown onset (=wake‐up)	−0.09	0.91	0.73–1.13	0.398
Intravenous therapy	−0.29	0.75	0.57–0.98	0.038*
Vaccination status	0.00	1.00	0.75–1.33	0.989
Occlusion site M1	−0.38	0.68	0.52–0.89	0.005*
Occlusion site M2	0.12	1.13	0.85–1.5	0.411
Occlusion site M3	0.10	1.11	0.76–1.59	0.605
Occlusion site A1	0.21	1.23	0.78–1.95	0.364
Occlusion site BA	−0.12	0.89	0.65–1.2	0.427
Occlusion site 9	0.12	1.13	0.75–1.7	0.557
Acute COVID‐19 infection	0.36	1.43	1.07–1.91	0.016*
Respiratory status in patients with acute COVID‐19 (none vs ventilation)	0.19	1.21	0.94–1.54	0.137
Respiratory status in patients with acute COVID‐19 (none vs intubation)	0.30	1.35	1.08–1.69	0.010*
TOAST cardioembolic	0.08	1.08	0.84–1.38	0.550
TOAST small‐vessel occlusion	−0.11	0.90	0.63–1.26	0.519
TOAST other	−0.06	0.94	0.66–1.34	0.723
TOAST undetermined	0.02	1.02	0.76–1.35	0.913
Tandem occlusion	0.18	1.20	0.9–1.6	0.225
Baseline ASPECTS	−0.19	0.83	0.69–0.98	0.028*
NIHSS admission score	0.04	1.04	0.98–1.11	0.159
mRS score pretreatment	0.21	1.23	1.05–1.46	0.010*
Final TICI (2a)	0.18	1.20	0.9–1.58	0.220
Final TICI (2b)	−0.09	0.91	0.71–1.19	0.513
Final TICI (2c)	0.04	1.04	0.79–1.39	0.761
Final TICI (3)	−0.20	0.82	0.64–1.06	0.131
Final TICI (1)	−0.27	0.76	0.58–1	0.054
Time from groin puncture to final recanalisation time	0.00	1.00	0.99–1.01	0.860
Anesthesia (analgosedation)	0.19	1.21	0.86–1.69	0.271
Complications (SAH)	−0.02	0.98	0.72–1.34	0.902
Complications (sICH)	0.21	1.23	0.92–1.66	0.158
Complications (SAH+sICH)	0.12	1.13	0.8–1.58	0.508
Other complications	0.07	1.07	0.77–1.5	0.674
Atrial fibrillation	0.14	1.15	0.88–1.5	0.317
Arterial hypertension	−0.05	0.95	0.72–1.26	0.732
Diabetes	0.02	1.02	0.76–1.37	0.882
Dyslipidemia	−0.26	0.77	0.57–1.03	0.080
Smoker	−0.01	0.99	0.72–1.35	0.944

Aikaike information criterion=112.9, sample size=118 (favorable outcome: 92; unfavorable outcome: 26). ASPECTS indicates Alberta Stroke Program Early CT [Computed Tomography] Score; BA, basilar artery; mRS, modified Rankin Scale; NIHSS, National Institutes of Health Stroke Scale; OR, odds ratio; SAH, subarachnoid hemorrhage; sICH, symptomatic intracranial hemorrhage; TOAST, Trial of Org 10172 in Acute Stroke Treatment; TICI, Thrombolysis in Cerebral Infarction. *P<0.05.

## Discussion

In this multicenter, retrospective analysis, we observed no significant difference in angiographic and clinical outcomes between vaccinated and unvaccinated patients. The rates of successful and complete final reperfusion in both groups were high (mTICI ≥2b: 87% NoVAX and 85% VAX; mTICI 3: 44% NoVAX and 40% VAX) and comparable to other MT studies during and before the pandemic.^4^, ^11^, ^12^ In our study, we could not observe an effect of vaccination on recanalization; in particular, we found no evidence that vaccination reduced clot burden.^13^ The 6 patients who received the Ad26.COV‐2.S vaccine (Johnson & Johnson/Janssen) and the ChAdOx1‐S vaccine (AstraZeneca), both vaccines based on an adenoviral vector, did not exhibit the prothrombotic state of vaccine‐induced immune thrombotic thrombocytopenia. Nevertheless, vaccine‐induced immune thrombotic thrombocytopenia, characterized by thrombosis at atypical sites combined with thrombocytopenia, has been observed in individuals after vaccination with the 2 previously mentioned adenoviral vector‐based agents and appears to be exceedingly rare following the vaccination of >400 million people worldwide.^14^

Our retrospective analysis revealed a higher rate of sICH in the VAX group compared with the NoVAX group (15% VAX versus 5% NoVAX; *P*=0.05). Consistent with this, a higher rate of IVT was observed in vaccinated patients. A systematic review analyzing thromboembolic and bleeding events after vaccination against SARS‐CoV‐2 demonstrated no increased risk of hemorrhage and death from thromboembolism and hemorrhage after vaccination against SARS‐CoV‐2 across all vaccine platforms.^15^ Therefore, it is unlikely that the high rate of sICH in our study was related to the vaccination status, and it is more likely that other confounders (such as IVT) contributed.

Most important, our study demonstrated that vaccinated and unvaccinated patients with COVID‐19 and AIS had similar devastating outcomes, although the reperfusion rates were high. The VAX group showed a slightly higher rate of functional independence (mRS score, ≤2) and a higher median NIHSS score at discharge compared with the NoVAX group, although without statistical significance. Consistent with a previous study of a large series of patients with COVID‐19 treated with MT because of large‐vessel occlusion, the mortality rate was high, up to 30%.^4^ Compared with studies with non–COVID‐19 populations, the mortality in our study is twice as high as in the meta‐analysis from Highly Effective Reperfusion Evaluated in Multiple Endovascular Stroke (15%). To date, few studies have focused on the effect of vaccination status in patients with stroke with or without the additional diagnosis of COVID‐19 infection.^16^, ^17^ El Naamani et al reported rates of functional independence (mRS score, ≤2) of 30.6% in the NoVAX group and 45.4% in the VAX group (*P*=0.044) of 203 COVID‐19–positive patients with stroke. Mortality was 20.2% in unvaccinated individuals and 7.8% in vaccinated individuals (*P*=0.033). The authors discuss that the administration of the vaccine and the consequent modulation of the immune system by minimizing the prothrombotic and proinflammatory milieu of COVID‐19 may reduce the severity of stroke and, therefore, may be the basis for the improved outcome of vaccinated patients.^16^, ^17^ It remains unknown why our study could not find a significant difference in short‐term clinical outcome between the 2 groups. It is likely that significant differences in baseline characteristics, such as the significantly higher rate of M2 occlusions in the NoVAX group, may affect the outcome. However, all factors considered, acute COVID‐19 infection proved to be an independent factor for unfavorable outcome in multivariable analysis, as previously demonstrated in other studies.^18^, ^19^ Therefore, the distinction between acute and subsided COVID‐19 infection is critical in terms of its impact on clinical outcome.

The main limitations of our study are the retrospective and multicenter design, including attendant selection bias and bias attributable to different COVID‐19 waves with correspondingly different severity of disease progression. The sample size limits the conclusions of these data. Although we used a robust multivariable model, it can only test for association and not directly for causality. Therefore, a careful prospective study using more elaborate modeling should be conducted in a future study. In addition, we did not correct for multiple testing, which could lead to inflation of the type 1 error rate. Furthermore, no formal sample size or power analysis was performed.

In addition, patients not receiving IVT had worse outcome, but it is likely that the results observed here represent confounding by indication that patients with IVT contraindications are more likely to have serious comorbidities and, therefore, worse outcomes.

Nevertheless, vaccination remains the safest strategy for avoiding hospitalizations, long‐term health outcomes, and death. This general health care recommendation remains untouched by the present results.

## Conclusions

In our study, we did not observe an impact of COVID‐19 vaccination on the angiographic or clinical outcome of COVID‐19–positive patients with AIS undergoing MT. Moreover, we found similarly devastating outcomes with high rates of mortality in vaccinated and unvaccinated patients with stroke.

## Sources of Funding

We acknowledge support by the Open Access Publication Fund of the University of Duisburg‐Essen.

## Disclosures

None.
